# An optimized microarray platform for assaying genomic variation in *Plasmodium falciparum *field populations

**DOI:** 10.1186/gb-2011-12-4-r35

**Published:** 2011-04-08

**Authors:** John C Tan, Becky A Miller, Asako Tan, Jigar J Patel, Ian H Cheeseman, Tim JC Anderson, Magnus Manske, Gareth Maslen, Dominic P Kwiatkowski, Michael T Ferdig

**Affiliations:** 1 The Eck Institute for Global Health, University of Notre Dame, 100 Galvin Life Sciences, Notre Dame, IN 46556, USA; 2 Roche NimbleGen Inc., 504 South Rosa Road, Madison, WI 53719, USA; 3 Department of Genetics, Texas Biomedical Research Institute, 7620 NW Loop 410, San Antonio, TX 78245, USA; 4 Wellcome Trust Sanger Institute, Wellcome Trust Genome Campus, Hinxton, Cambridge CB10 1SA, UK; 5 Current address: 100 Galvin Life Sciences, Notre Dame, IN 46556, USA

## Abstract

We present an optimized probe design for copy number variation (CNV) and SNP genotyping in the *Plasmodium falciparum *genome. We demonstrate that variable length and isothermal probes are superior to static length probes. We show that sample preparation and hybridization conditions mitigate the effects of host DNA contamination in field samples. The microarray and workflow presented can be used to identify CNVs and SNPs with 95% accuracy in a single hybridization, in field samples containing up to 92% human DNA contamination.

## Background

*Plasmodium falciparum *is the intracellular parasite responsible for the majority of the world's malaria morbidity and mortality burden in humans, causing an estimated 243 million episodes of malaria and 863,000 deaths each year [1]. Efforts to control and eradicate malaria are hampered by the accelerated evolution of drug resistance in the parasite. To date, the parasite has developed resistance to all major antimalarial drugs, raising concerns about the spread of drug-resistant parasites and the ability to effectively treat malaria [2]. The development of new technologies aimed at understanding parasite genome variability provides hope in identifying new drug targets, implementing smarter treatment plans, and ultimately reducing or eliminating the burden of malaria.

Genome variation such as SNPs and copy number variation (CNV) underpins *P. falciparum *drug resistance. The primary determinant of chloroquine resistance is a mutation in the *P. falciparum *chloroquine resistance transporter gene on chromosome (chr) 7 [3,4]. *In vitro *resistance to the antifolate drugs sulfadoxine and pyrimethamine increases in a step-wise manner as mutations accrue in dihydrofolate reductase and dihydropteroate synthase [5-8]. Varying copy number of the *P. falciparum *multidrug resistance 1 gene on chr 5 influences parasite susceptibility to a range of antimalarial drugs, including mefloquine, lumefantrine, quinine, and artemisinin [9-11]. Amplification on chr 12 of GTP cyclohydrolase 1 of the folate biosynthesis pathway is correlated with antifolate drug resistance [12,13]. These examples emphasize the importance of genomic variation in drug resistance and need to assay both SNPs and CNV genome-wide in the malaria parasite.

Microarrays provide a relatively fast and inexpensive way of examining genomic variation in *P. falciparum *[14]. Array comparative genomic hybridization (CGH) has been successfully used to look at structural variation and CNV in multiple *P. falciparum *strains [12,15-17], while large-scale sequencing efforts identifying SNPs [18-20] have spurred the development of SNP microarrays. Neafsey *et al. *[21] genotyped 1,638 out of 3,000 queried SNPs with 100% accuracy using an Affymextrix 3K SNP assay. Mu *et al. *[22] used Affymetrix molecular inversion probe technology to genotype 2,763 of 3,354 SNPs with >90% call rate. Multiple groups have successfully applied CGH for SNP detection with 80 to 90% sensitivity to approximately 3,000 SNPs [23,24] and identified parameters influencing SNP detection [24,25]. However, the reported detection rates are based on a core subset of SNPs (approximately 3,000) in a genome with more than 100,000 cataloged SNPs (PlasmoDB v5.5) [26].

**Figure 1:**
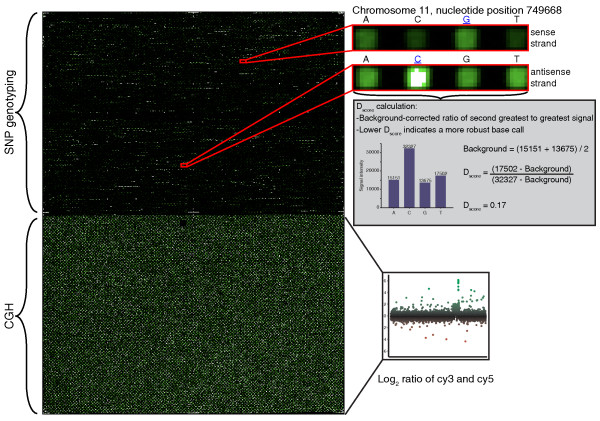
**Microarray layout and design**. The microarray contains blocks of probesets for SNP genotyping and CGH. SNP genotyping probesets are composed of two probe quartets, one for each strand (red blowouts). Probes from one quartet typically have hybridization signals in a similar dynamic range. These signals are used to determine a base call and calculate base calling robustness, which is expressed as a *D*_*score *_where the two lesser hybridization signals are used to estimate background noise for each probe quartet (grey inset). The CGH probesets record data that are used to generate log_2 _ratios of a test and reference sample.

Our central goal was to develop a single microarray platform that can assay CNV and genotype SNPs simultaneously and to optimize this platform for the challenges of monitoring monoclonal patient blood samples from field studies. We first empirically determined the optimum probe lengths and melting temperatures for SNP genotyping in the 81% AT *P. falciparum *genome. This was used to guide the design of a single high-resolution genotyping microarray with variable length probes optimized for high quality SNP genotyping and CNV detection (Figure 1). One half of the microarray interrogates 45,524 SNP loci using optimized resequencing probes 29 to 41 bp in length capable of making a base call at a precise nucleotide position [27]. The second half identifies CNV using tiled CGH probes 50 to 75 bp in length. We determine the reliability and accuracy of the CNV-SNP array using the laboratory lines 3D7, HB3, Dd2, SC05, and 7C126. We then validate the utility and robustness of the microarray using field samples with limited parasite DNA and high human DNA contamination present using blood collected from humans at the Thailand-Burma border.

## Results

### Effects of probe length and probe melting temperature on the robustness of base calls

**Figure 2:**
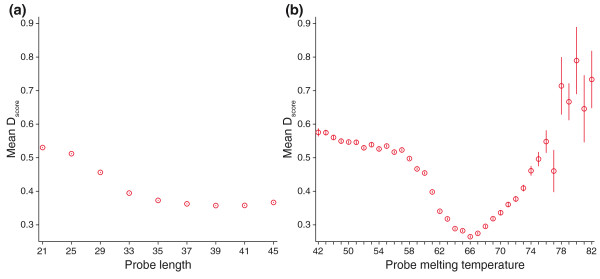
**Base calling robustness is affected by probe length and melting temperature**. Mean *D*_*score *_is plotted by **(a) **probe length and **(b) **probe melting temperature where vertical lines indicate 95% confidence intervals. Lower discrimination scores indicate greater base calling robustness. Fixed length 39-mers provided the best performance for any static probe length tested. Probes with a 66°C melting temperature provided the best performance for any melting temperature range and surpassed the performance of 39-mer probes.

Using a prototype 5K SNP chip, the performance of static probe lengths was compared to the performance of variable length, isothermal probes on base calling robustness. A base call is considered robust when a probe quartet has a single high nucleotide signal relative to the other three nucleotide signals, and the sense and antisense base calls are complementary. A *D*_*score *_calculating the background noise relative to the highest signal intensity in a probe quartet (Figure 1, grey inset) was used to compare the performance of static probes and isothermal probes. A *D*_*score *_close to 1 indicates high background noise and poor discrimination ability between probe signals, while a score close to 0 indicates low background noise and good discrimination ability. The mean *D*_*score *_of the 5K SNP chips was plotted for static probe lengths (Figure 2a) and for probe melting temperature (Figure 2b). Statistical analysis with a one-way ANOVA indicates significant differences between mean *D*_*score *_at various probe lengths (*P *< 0.0001), and a Tukey's multiple comparison test indicates that all probe lengths except 39- and 41-mers have a significantly different mean *D*_*score *_(*P *< 0.05). Out of the nine tested probe lengths, 39-mer probes generated the lowest mean *D*_*score*_ with the best discrimination ability ( = 0.3575). A one-way ANOVA analysis indicates probe melting temperature *D*_*score *_are significantly different from one another (*P *< 0.0001), and a Tukey's multiple comparison test indicates melting temperature in the 66°C range was significantly different from other melting temperatures (*P *< 0.05). The lowest mean *D*_*score *_with the best discrimination ability for probe melting temperatures was generated at 66°C from the range of 42 to 82°C ( = 0.2647), and this outperformed any static probe lengths. Similar performance was seen when comparing exons, introns, and intergenic regions (Figure S1 in Additional file 1).

### Microarray base calling accuracy

**Figure 3:**
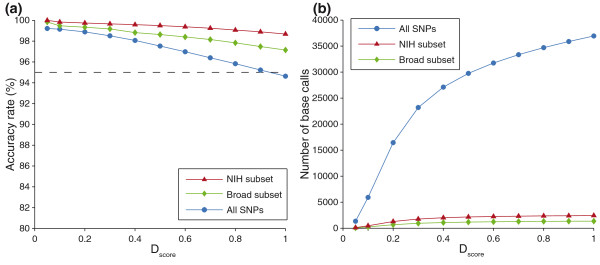
**Microarray accuracy rate and base calling**. **(a) **Microarray base calling accuracy rate was calculated by comparing microarray base calls with sequence data for five parasite genomes. At more stringent discrimination scores, accuracy rate increased. SNP subsets demonstrate greater performance than average if those SNPs are more amenable to hybridization-based interrogation (NIH and Broad subsets). **(b) **Mean number of base calls produced at various *D*_*score *_cutoffs for all SNP loci and for SNP subsets from previously published microarray platforms (NIH and Broad).

Microarray data for 3D7, HB3, Dd2, SC05, and 7C126 (n = 15, 5, 3, 2, and 2, respectively) were compared to genome sequence data to ascertain base calling accuracy (Figure 3a). A useable base call was made at a SNP locus when the sense and antisense probesets indicated complementary bases; otherwise, there would be no base call for that SNP locus. Figure 3a depicts mean microarray accuracy plotted by *D*_*score*_; also depicted are CNV-SNP array data for the SNP subsets that are represented on SNP genotyping microarrays developed by the Broad Institute [21] and NIH [22]. The CNV-SNP array genotypes 1,507 of the 1,631 publicly available Broad Institute SNPs and 2,621 of the 2,743 publicly available NIH SNPs. For all SNP sets, a lower *D*_*score *_is associated with higher accuracy. SNPs from the Broad Institute and NIH maintain >95% accuracy at all *D*_*score *_cutoffs with 97.1% accuracy and 98.7% accuracy, respectively, at a *D*_*score *_cutoff of 1. The accuracy of all SNPs assayed on the CNV-SNP array maintain >95% accuracy for *D*_*score *_≤0.9 but drops to an average accuracy of 94.6% at a *D*_*score *_of 1. Figure 3b depicts the mean number of base calls made by the microarray plotted by *D*_*score *_for all SNPs and for the SNP subsets from the Broad Institute and NIH microarray platforms (Figure 3b). On average, a microarray hybridization yielded 36,948 base calls from the 45,524 assayed SNP loci for a base call rate of 81.2%. The Broad Institute and NIH SNP subsets had an average call rate of 90.7% and 93.0%, respectively. Lower numbers of base calls are made at more stringent *D*_*score *_cutoffs for all data sets. When comparing accuracy for SNP subsets in exons, introns, and intergenic regions, introns and intergenic regions perform similarly, and exons exhibited the best performance (Table 1).

**Table 1:** Microarray accuracy in exons, introns, and intergenic regions

Location	**Accuracy, *D***_***score ***_**≤1.0**	**Accuracy, *D***_***score ***_**≤0.5**
Exon	96.3%	98.5%
Intron	91.2%	95.4%
Intergenic region	90.4%	94.9%

### Comparative genomic hybridization performance

**Figure 4:**
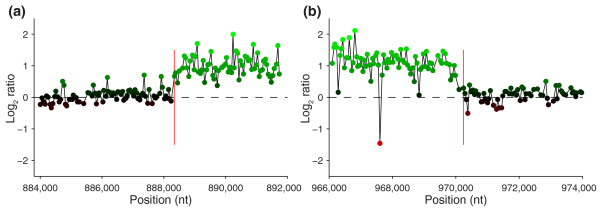
**CNV breakpoint detection with CGH**. CGH data for a CNV on chr 5 in the Dd2 genome accurately detects the breakpoints for the **(a) **beginning and **(b) **end of the event within hundreds of base pairs. The vertical red lines indicate the breakpoint locations as previously determined through sequence data [43]. Nt, nucleotides.

Segmentation analyses on multiple hybridizations of HB3 and Dd2 against the reference 3D7 detected the same copy number events detected in HB3 and Dd2 hybridizations against 3D7 found previously [25]. Median probe spacing between the 5' end of CGH probes is 52 bp, providing a fine resolution view of CNV that can precisely implicate breakpoints to within 100 bp. The resolution provided by this platform is equivalent to a previous NimbleGen CGH chip [25]. Figure 4 shows CGH scatterplots demonstrating microarray-based CNV breakpoint detection in comparison with the exact breakpoint determined through capillary sequencing.

CNV events are highly reproducible between replicate hybridizations with this microarray platform (Figure S2 in Additional file 1). Features, including a 500 bp CNV event, were recognized and consistent between hybridizations; however, it becomes more difficult to confidently detect small CNV events algorithmically. CNV event detection is still possible with whole genome amplification (WGA) samples (Figure S3 in Additional file 1), although the amplification process introduces noise, confounding CNV detection by any platform, particularly reducing confidence in small events. Degraded samples can not be recovered for effective CGH by WGA.

### Applications to *P. falciparum *field samples

Standard probe labeling protocols utilize random nonamers with balanced base composition. However, *P. falciparum *has an extremely high AT content of 81% [28], which reduces the performance of 50% AT random nonamers and may introduce bias during amplification of the parasite genome. To test the effect of random nonamer AT composition on labeling performance and microarray data, labeling yields of 65% AT random nonamers (38,357 ± 3468.1 ng) were compared with labeling yields of 50% AT random nonamers (18,865 ± 4530.7 ng). The data pass a D'Agostino-Pearson normality test and a paired *t*-test, indicating that 65% AT random nonamers generate a significantly greater yield of labeled DNA than 50% AT random nonamers (*P *< 0.01). We see no adverse effects on base calling accuracy or CGH performance when using this modified labeling procedure.

**Figure 5:**
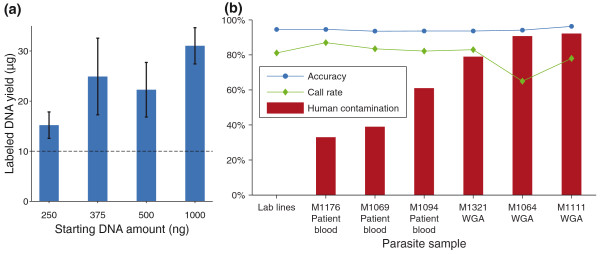
**Field sample analysis with the CNV-SNP array**. **(a) **The manufacturer-recommended starting amount is 1,000 ng of DNA to produce at least 10 μg of labeled product. However, 250 ng of parasite DNA consistently produced sufficient labeled product when using 65% AT nonamers. Error bars indicate one standard deviation. **(b) **Hybridizations with field samples - straight from patient blood, or whole genome amplified - produced microarray data on par with standard lab clones, even when significant human DNA contamination was present. Microarray accuracy was determined through Illumina sequencing of lab-adapted parasites. Patient blood samples were hybridized with the addition of 1× Denhardt's solution while WGA samples were not.

Larger yields of labeled DNA are generated by 65% AT random nonamers, allowing the amount of initial starting DNA to be reduced. We evaluated decreasing amounts of starting material that could generate the necessary 10 μg of labeled DNA for hybridization. Labeling yields using 250 ng, 375 ng, 500 ng, and 1,000 ng of starting DNA for 3D7, HB3, and Dd2 were quantified (Figure 5a) and hybridized on the microarray. More than 10 μg of labeled DNA was obtained from all labeling reactions and no base calling or CNV detection differences were seen between the different starting amounts, indicating a starting DNA amount ≤250 ng is sufficient to generate high quality hybridization data.

Human DNA is invariably present in field-collected samples of parasite DNA and is especially high when leukocyte depletion is not used in the extraction method. In some cases, human DNA may constitute >90% of the total DNA extracted from infected blood samples and can hinder downstream uses of the parasite DNA for microarray hybridizations or sequencing. Nucleic acid blockers are commonly used in microarray hybridizations to prevent random probe binding to non-target nucleic acids and were tested on our microarray to prevent performance reduction in samples with significant amounts of human DNA contamination. We tested various nucleic acid blockers and found that 1× Denhardt's solution provided the greatest number of base calls with no negative impact on CGH data when compared to hybridizations with bovine serum albumin, dimethyl sulfoxide, human Cot1 DNA, salmon sperm, and yeast tRNA. To test for the effect of human DNA contamination on our microarray, several DNA samples were extracted from Thailand-Burma patient blood samples or were whole genome amplified from patient blood. The amount of human DNA present in each sample was quantified, and 33 to 92% of the total DNA was found to be human, with WGA samples containing the most human DNA. WGA is generally, but not always, helpful as there is some variability in data generated from WGA samples. Using 250 ng of parasite DNA, field samples were hybridized to the microarray and examined for the number and accuracy of base calls (Figure 5b); accuracy was determined using Illumina sequences generated from the same samples (M Manske, unpublished observations). This platform is able to produce high quality data with samples containing extensive host DNA contamination equivalent to data from purified lab line DNA (Figure 5b).

## Discussion

The CNV-SNP array provides robust, accurate data for both laboratory- and field-derived samples. Through optimizations described here, the CNV-SNP array overcomes many hurdles associated with molecular work on *P. falciparum *field samples. Lower starting amounts of DNA are possible when using 65% AT random nonamers that compensate for the extreme AT bias of the genome. This optimization is especially useful for field sample DNA, which is typically scarce and difficult to obtain. It also eliminates the need for *in vitro *culture adaptation of field samples, which is typically used to generate enough DNA for applications like next-generation sequencing and is known to alter CNV and skew results of CNV analyses [29,30]. Using our modified protocol, the CNV-SNP array requires no more than 250 ng of starting parasite DNA with no compromise in data quality. Moreover, the ample yields of labeled DNA from 250 ng of starting parasite DNA indicate that the lower limit has not yet been defined, raising the possibility that finger prick blood samples on filter paper are accessible to this technology. In addition, the CNV-SNP array is robust to samples with high host DNA contamination (>90%) with no drop in data quality, making microarray-based genotyping complementary to higher resolution next-generation sequencing that is sensitive to human DNA contamination in field samples, often requiring sample preprocessing for target DNA enrichment. Notably, high human DNA contamination and low amounts of parasite DNA present serious challenges to genotyping the large number of samples necessary for genome-wide association studies.

Probe design optimizations contribute to the performance of this microarray for the *P. falciparum *genome. Sense and antisense resequencing probe quartets were used for SNP genotyping on the CNV-SNP array. A SNP call required that sense and antisense probe quartets made complementary calls; furthermore, the robustness of the base call was evaluated using the ratio of background signal versus the probe with the greatest signal intensity. Signal intensities within SNP probe quartets were more similar to each other than to probes in other probe quartets or between sense and antisense probe quartets of the same locus (Figure 1) and indicates the importance of measuring the background signal for each individual SNP quartet - as provided by the resequencing probesets - rather than background noise from the entire array or locus.

Resequencing probes were optimized for SNP genotyping in *P. falciparum *by comparing the performance of probes at static lengths with probes balanced by melting temperature on a prototype 5K SNP array. Probe melting temperature outperformed static probe lengths for optimal SNP detection at a probe melting temperature of 66°C with performance that was reasonably consistent in exons, introns, and intergenic regions (Figure S1 in Additional file 1).

Our results on optimal probe length and melting temperature differ from findings in another study [31]. This is likely due to the use of different methods for calculating probe melting temperature and our optimization to the AT-rich *P. falciparum *genome. However, our broader conclusion that variable length or isothermal probes provide optimal SNP detection is supported across various organisms [31,32], and indicate that longer, isothermal probes increase signal strength while also being short enough to remain sensitive to single base mismatches [32-35].

Resequencing probesets designed for a 66°C melting temperature were generated for 45,524 SNP loci for inclusion on the CNV-SNP array. While longer, isothermal probes improve SNP genotyping, certain loci are more easily genotyped than others, and some remain inaccessible to microarrays and short-read next-generation sequencing technologies. For instance, SNPs in exons have greater genotyping success than SNPs in introns or intergenic regions, likely due to regions of high AT richness or interspersed sequence repetitiveness that hinder probe design and binding specificity in intronic and intergenic regions. Current SNP genotyping microarrays, such as those developed by the NIH and the Broad Institute [21,22], are focused on high quality SNP loci that are easily genotyped across microarray platforms (Figure 3). However, the use of isothermal probes designed at an optimal melting temperature allows us to interrogate more difficult loci and maximize the overall number of SNPs that can be robustly genotyped on the CNV-SNP array (on average, 36,948 useable SNP genotypes with 95% accuracy from a single hybridization).

An interesting debate surrounds the continued value of microarrays with the emergence of next-generation sequencing. As the cost of next-generation sequencing continues to decrease and protocols continue to improve, we will see a realization of the platform providing ultimate resolution and throughput, provoking the prediction that microarrays will soon be rendered obsolete. However, we suggest that the CNV-SNP array will continue to be useful as an 'everylab' tool alongside next-generation sequencing. Whole genome sequencing underpins the SNP discovery needed for chip design; in general, whole genome architecture and ultra-resolution mapping require fully sequenced and assembled genomes. The customizable microarray platform continues to improve in density (4.2 million element custom designs are anticipated in 2011) and offers unique configurations up to 12-plex of 135K probes, leading to a scenario in which a global set of SNPs identified by sequencing can be precisely represented on microarrays for regionally focused or hypothesis-driven designs. To date, microarrays remain cheaper, produce data more quickly, require less computational innovation, and are especially useful for processing large numbers of samples, while producing sufficient resolution and quality for genome-wide association studies and population genomic analysis. Furthermore, although progress is being made in scoring CNV using next-generation sequencing, that technology still lags behind the performance of microarray CGH.

## Conclusions

As *P. falciparum *continues to evolve and evade control and eradication efforts, high-throughput, cost-effective methods of monitoring genomic variation are critical to understanding parasite adaptation. The high AT content of the *P. falciparum *genome is technically challenging for most molecular methods; however, the flexibility of the microarray platform described here allows users to customize and optimize microarrays to individual genomes through alterations of probe lengths, types, and numbers and adjustment of hybridization strategy. This process is applicable to population genomic studies in a wide range of organisms. Utilizing this flexibility, we created a custom high-density CNV-SNP array containing both resequencing probes capable of SNP genotyping and CGH probes for CNV detection. The CNV-SNP array is a reliable, accurate platform that allows simultaneous investigation of CNV and SNPs in a single hybridization. Its low cost, quick turn-around time, low DNA requirements, and resilience to human DNA contamination make it a valuable tool for population genomic studies.

## Materials and methods

### Microarray design

#### Probe length optimization

As an initial step in SNP genotyping optimization, a NimbleGen resequencing microarray consisting of variable length resequencing probesets was developed to assay 5,347 SNP loci. A NimbleGen resequencing probeset is composed of eight probes per SNP locus: four probes each for interrogation of sense and antisense strands. Each probe quartet is identical except for the central nucleotide that assays the nucleotide variant [27]. We downloaded 101,581 candidate SNP loci from PlasmoDB v5.5 [26] for parasite isolates HB3, Dd2, V1/S, 7G8, D10, FCC-2, K1, RO-33, D6, GHANA1, FCB, and IT. Common SNPs (SNPs identified in at least two parasite isolates) were blasted against the 3D7 genome to verify a unique 21-mer SNP typing probe sequence. Probesets with more than one exact match in the genome were discarded. Of the remaining candidate probesets, 5,347 SNP loci were randomly chosen for inclusion on the 5K SNP chip at nine different probe lengths: 21, 25, 29, 33, 35, 37, 39, 41, and 45-mers. Hybridizations following NimbleGen standard CGH procedures [36] were performed using DNA from laboratory clones 3D7, HB3, Dd2, 7G8, and D10.

#### CNV-SNP array

Using NimbleGen's 3-plex custom chip layout, each plex in our 3-plex 720K NimbleGen microarray contains resequencing probes for SNP genotyping and CGH probes for CNV detection (Figure 1). Probes were synthesized using maskless photolithography [37,38] with CGH probes attached by 5T linkers and resequencing probes attached with 15T linkers. Of the 101,581 candidate SNP loci downloaded from PlasmoDB and BLASTed for uniqueness, probesets for all SNPs reported in at least two parasites lines were included in the microarray. Some SNPs represented by a single isolate were included on the array and prioritized by mutation types sensitive to array detection [25]. In total, 45,524 SNP loci queried by 364,192 probes balanced to 66°C melting temperature were included on the microarray. CGH microarray probes were designed using standard NimbleGen CGH protocol [36] modified for the *P. falciparum *genome. Briefly, probes were tiled through the genome at 4-bp interval spacing and filtered for 60 to 80°C melting temperature and 50 to 75 bp length. The resulting probes were clustered with nearest neighbors and sorted to remove probes with extensive sequence identity to any other probe. Probes with more than one 50-mer exact match in the genome or located in hypervariable *var*/*rif*/*stevor *genes were discarded. The median spacing between the start of the 355,803 CGH probes is 52 bp.

### Parasite samples

Fresh cultures of cloned 3D7, HB3, Dd2, SC05, and 7C126 lines derived from genotype-confirmed stock material were grown under standard cultivation conditions [39,40]. Parasite DNA was extracted using standard phenol/chloroform extraction and concentrated by salt precipitation [39]. Parasite DNA from the Thailand-Burma border was collected from 5 ml whole blood of symptomatic patients visiting malaria clinics. Buffy coats from the blood samples were removed and infected red blood cells cultured for 24 h to allow ring stage parasites to mature to schizonts to provide more DNA. DNA was extracted using a standard phenol/chloroform protocol. Parasite isolates were screened for multiple infections and identical clones using seven polymorphic microsatellite markers: *ARA2 *(chr 11), *POLYα *(chr 4), *TA1 *(chr 6), *C2M1 *(chr 2), *C3M54 *(chr 3), *TA60 *(chr 13), and *C4M30 *(chr 4). These markers were amplified using fluorescent end-labeled oligos and run on an ABI 3100 capillary sequencer (Applied Biosystems Inc., Foster City, CA, USA) and alleles scored using GeneScan (Applied Biosystems Inc.) and Genotyper (Applied Biosystems Inc.) software. Samples were considered multiple clone infections if one or more of the seven microsatellite loci showed multiple alleles. Only unique genotypes were included in the study. Thailand-Burma samples with an inadequate amount of DNA were whole genome amplified using phi29 DNA polymerase (Fidelity Systems, Gaithersburg, MD, USA). WGA samples were cleaned using a standard phenol/chloroform protocol. Parasite DNA concentrations in the Thailand-Burma samples were measured using quantitative PCR. Patient samples were amplified using SYBRgreen (Applied Biosystems Inc.) with primers specific to the *P. falciparum ama1 *gene. Reactions were run on an ABI PRISM 7900HT realtime PCR machine and DNA amounts calculated by comparison with a dilution series of pure DNA from parasite line 3D7. DNA from lab-adapted samples was submitted to the Wellcome Trust Sanger Institute (Hinxton, UK) for Illumina sequencing using 76-bp paired-end reads.

### Microarray hybridizations

Labeling and hybridization were conducted using standard NimbleGen CGH procedures [36]. gDNA (250 ng to 1 μg) was denatured at 98°C for 10 minutes in the presence of 1 OD of cy3 or cy5-labeled random nonamers at 50% AT richness or 65% AT richness (TriLink Biotechnologies, San Diego, CA, USA). The denatured sample was quick chilled on ice and incubated with 50 units of Klenow fragment (New England Biolabs, Ipswich, MA, USA) and dNTP mix (6 mM each in TE (Sigma Aldrich, St Louis, MO, USA)) for 2 h at 37°C. Reactions were terminated with 0.5 M EDTA and precipitated with 5 M NaCl in isopropanol. Labeled product was resuspended in water, and 10 μg of test and reference samples combined (6 μg for 5K SNP chip samples), dried down, and resuspended in hybridization buffer (Roche NimbleGen, Inc., Madison, WI, USA); hybridizations for patient blood samples included 1× Denhardt's solution (Sigma Aldrich) in the hybridization buffer. The combined sample was denatured at 95°C for 5 minutes and allowed to hybridize on the array for 24 h (16 h for 5K SNP chip samples) at 42°C in a NimbleGen hybridization system (Roche NimbleGen, Inc.). Microarrays were washed sequentially in Wash Buffer I (2 minutes at room temperature), Wash Buffer II (1 minutes at room temperature), and Wash Buffer III (15 s at room temperature; Roche NimbleGen, Inc.) and dried for 1 minuets in a Microarray High-Speed Centrifuge (Arrayit Corp., Sunnyvale, CA, USA). CNV-SNP arrays were scanned at 2 μm resolution using a NimbleGen MS 200 Microarray Scanner (Roche NimbleGen, Inc.). 5K SNP chips were scanned at 5 μm resolution using a GenePix Pro 4200A Scanner (Molecular Devices, Inc., Sunnyvale, CA, USA). Microarray data are deposited at Gene Expression Omnibus, accession number [GEO:GSE28287].

### Microarray data analysis

#### SNP analysis

Data for 3D7, HB3, Dd2, SC05, and 7C126 were extracted from scanned images and resequencing base reports generated using NimbleScan v2.5 (Roche NimbleGen, Inc.). Base calls were made on resequencing probesets when the sense and antisense probes made complementary base calls. The discrimination score (*D*_*score*_) for each probe quartet was calculated as a background corrected ratio of the signal from the second greatest intensity probe from a probe quartet divided by the greatest intensity probe using custom perl scripts (Figure 1, grey insert): (Second highest signal intensity - Background)/(Highest signal intensity - Background). Background for each probe quartet was calculated as the average of the third and fourth highest signal intensities [32]. There is some noise inherent in this base calling method, which can be mitigated by performing technical replicates or incorporating probe replicates into the chip design. For SNP genotyping probes, probeset melting temperature was calculated as previously described [36] where mean melting temperature was calculated from all probes in each probe quartet. The accuracy of the base calls made by the resequencing probes was calculated as the percentage of base calls that matched the reference genome for 3D7 or draft genome assemblies for HB3, Dd2, SC05, and 7C126 [20,28,41]. To ascertain the base called at a SNP locus in the genome assembly, resequencing probes were mapped to draft genome assemblies using blastall, requiring at least 95% of the probe length with less than four mismatched/indel positions excluding the central nucleotide. Illumina sequence data were aligned to the 3D7 reference genome with SNP-o-matic software [42] to identify SNP locations for comparison to microarray data.

#### Copy number variation analysis

Data for HB3 and Dd2 (n = 6 and n = 4, respectively) hybridized against 3D7 were extracted from scanned images and normalized using NimbleScan v2.5 (Roche NimbleGen, Inc.). Copy number events from the segmentation analysis in HB3 and Dd2 against the reference 3D7 were compared to known CNV in the published literature [25].

## Abbreviations

Bp: base pair; CGH: comparative genomic hybridization; chr: chromosome; CNV: copy number variation; SNP: single nucleotide polymorphism; WGA: whole genome amplification.

## Authors' contributions

JCT conceived of the study, participated in its design and coordination, and drafted the manuscript. BAM participated in study design and coordination, performed microarray hybridizations, and drafted the manuscript. AT participated in microarray design and performed statistical analysis. IHC and TJA participated in study design and assisted with field samples. MM, GM, and DPK provided Illumina sequencing and data analysis. MTF conceived of the study, participated in its design and coordination, and helped to draft the manuscript.

## Supplementary Material

Additional file 1 **Supplementary Figures S1 to S3**. Figure S1: pptimal probe melting temperature is consistent in exons, introns, and intergenic regions. Mean *D*_*score *_is plotted by probe melting temperature in exons, introns, and intergenic regions; vertical lines indicate 95% confidence intervals. Probes with approximately 66°C melting temperature consistently provided the best performance. Figure S2: CGH data reproducibility. CGH scatterplots for individual CNV events are displayed for replicate hybridizations from independent labeling reactions demonstrating data reproducibility. CNV events from three separate parasite clones are displayed: **(a) **Dd2; **(b) **HB3; **(c) **SC05. The CNV breakpoints are precisely identified between hybridizations. Figure S3: CNV detection in a WGA field sample. CGH scatterplot for a CNV event detected in a WGA field sample, M1064. Four genes (PFE1150w, PFE1155c, PFE1160w, and PFE1165c) are affected by this CNV, including the *P. falciparum *multidrug resistance gene, *pfmdr1*. Click here for file

## References

[B1] WHO World Malaria Report 2009 2009 Geneva: World Health Organization

[B2] Enserink M Malaria's drug miracle in danger. Science 2010 328 844 846 10.1126/science.328.5980.844 20466917

[B3] Fidock DA Nomura T Talley AK Cooper RA Dzekunov SM Ferdig MT Ursos LM Sidhu AB Naude B Deitsch KW Su XZ Wootton JC Roepe PD Wellems TE Mutations in the *P. falciparum *digestive vacuole transmembrane protein PfCRT and evidence for their role in chloroquine resistance. Mol Cell 2000 6 861 871 10.1016/S1097-2765(05)00077-8 11090624 PMC2944663

[B4] Wellems TE Walker-Jonah A Panton LJ Genetic mapping of the chloroquine-resistance locus on *Plasmodium falciparum *chromosome 7. Proc Natl Acad Sci USA 1991 88 3382 3386 10.1073/pnas.88.8.3382 1673031 PMC51451

[B5] Lozovsky ER Chookajorn T Brown KM Imwong M Shaw PJ Kamchonwongpaisan S Neafsey DE Weinreich DM Hartl DL Stepwise acquisition of pyrimethamine resistance in the malaria parasite. Proc Natl Acad Sci USA 2009 106 12025 12030 10.1073/pnas.0905922106 19587242 PMC2715478

[B6] Mita T Origins and spread of pfdhfr mutant alleles in *Plasmodium falciparum*. Acta Trop 2010 114 166 170 10.1016/j.actatropica.2009.07.008 19607799

[B7] Plowe CV Cortese JF Djimde A Nwanyanwu OC Watkins WM Winstanley PA Estrada-Franco JG Mollinedo RE Avila JC Cespedes JL Carter D Doumbo OK Mutations in *Plasmodium falciparum *dihydrofolate reductase and dihydropteroate synthase and epidemiologic patterns of pyrimethamine-sulfadoxine use and resistance. J Infect Dis 1997 176 1590 1596 10.1086/514159 9395372

[B8] Triglia T Menting JG Wilson C Cowman AF Mutations in dihydropteroate synthase are responsible for sulfone and sulfonamide resistance in *Plasmodium falciparum*. Proc Natl Acad Sci USA 1997 94 13944 13949 10.1073/pnas.94.25.13944 9391132 PMC28412

[B9] Barnes DA Foote SJ Galatis D Kemp DJ Cowman AF Selection for high-level chloroquine resistance results in deamplification of the pfmdr1 gene and increased sensitivity to mefloquine in *Plasmodium falciparum*. EMBO J 1992 11 3067 3075 1353446 10.1002/j.1460-2075.1992.tb05378.x PMC556790

[B10] Cowman AF Galatis D Thompson JK Selection for mefloquine resistance in *Plasmodium falciparum *is linked to amplification of the pfmdr1 gene and cross-resistance to halofantrine and quinine. Proc Natl Acad Sci USA 1994 91 1143 1147 10.1073/pnas.91.3.1143 8302844 PMC521470

[B11] Sidhu AB Uhlemann AC Valderramos SG Valderramos JC Krishna S Fidock DA Decreasing pfmdr1 copy number in plasmodium falciparum malaria heightens susceptibility to mefloquine, lumefantrine, halofantrine, quinine, and artemisinin. J Infect Dis 2006 194 528 535 10.1086/507115 16845638 PMC2978021

[B12] Kidgell C Volkman SK Daily J Borevitz JO Plouffe D Zhou Y Johnson JR Le Roch K Sarr O Ndir O Mboup S Batalov S Wirth DF Winzeler EA A systematic map of genetic variation in *Plasmodium falciparum*. PLoS Pathog 2006 2 e57 10.1371/journal.ppat.0020057 16789840 PMC1480597

[B13] Nair S Miller B Barends M Jaidee A Patel J Mayxay M Newton P Nosten F Ferdig MT Anderson TJ Adaptive copy number evolution in malaria parasites. PLoS Genet 2008 4 e1000243 10.1371/journal.pgen.1000243 18974876 PMC2570623

[B14] Hayton K Su XZ Drug resistance and genetic mapping in *Plasmodium falciparum*. Curr Genet 2008 54 223 239 10.1007/s00294-008-0214-x 18802698

[B15] Carret CK Horrocks P Konfortov B Winzeler E Qureshi M Newbold C Ivens A Microarray-based comparative genomic analyses of the human malaria parasite *Plasmodium falciparum *using Affymetrix arrays. Mol Biochem Parasitol 2005 144 177 186 10.1016/j.molbiopara.2005.08.010 16174539

[B16] Cheeseman IH Gomez-Escobar N Carret CK Ivens A Stewart LB Tetteh KK Conway DJ Gene copy number variation throughout the *Plasmodium falciparum *genome. BMC Genomics 2009 10 353 10.1186/1471-2164-10-353 19653891 PMC2732925

[B17] Ribacke U Mok BW Wirta V Normark J Lundeberg J Kironde F Egwang TG Nilsson P Wahlgren M Genome wide gene amplifications and deletions in *Plasmodium falciparum*. Mol Biochem Parasitol 2007 155 33 44 10.1016/j.molbiopara.2007.05.005 17599553

[B18] Jeffares DC Pain A Berry A Cox AV Stalker J Ingle CE Thomas A Quail MA Siebenthall K Uhlemann AC Kyes S Krishna S Newbold C Dermitzakis ET Berriman M Genome variation and evolution of the malaria parasite *Plasmodium falciparum*. Nat Genet 2007 39 120 125 10.1038/ng1931 17159978 PMC2663918

[B19] Mu J Awadalla P Duan J McGee KM Keebler J Seydel K McVean GA Su XZ Genome-wide variation and identification of vaccine targets in the *Plasmodium falciparum *genome. Nat Genet 2007 39 126 130 10.1038/ng1924 17159981

[B20] Volkman SK Sabeti PC DeCaprio D Neafsey DE Schaffner SF Milner DA Jr Daily JP Sarr O Ndiaye D Ndir O Mboup S Duraisingh MT Lukens A Derr A Stange-Thomann N Waggoner S Onofrio R Ziaugra L Mauceli E Gnerre S Jaffe DB Zainoun J Wiegand RC Birren BW Hartl DL Galagan JE Lander ES Wirth DF A genome-wide map of diversity in *Plasmodium falciparum*. Nat Genet 2007 39 113 119 10.1038/ng1930 17159979

[B21] Dharia NV Sidhu AB Cassera MB Westenberger SJ Bopp SE Eastman RT Plouffe D Batalov S Park DJ Volkman SK Wirth DF Zhou Y Fidock DA Winzeler EA Use of high-density tiling microarrays to identify mutations globally and elucidate mechanisms of drug resistance in *Plasmodium falciparum*. Genome Biol 2009 10 R21 10.1186/gb-2009-10-2-r21 19216790 PMC2688282

[B22] Jiang H Yi M Mu J Zhang L Ivens A Klimczak LJ Huyen Y Stephens RM Su XZ Detection of genome-wide polymorphisms in the AT-rich *Plasmodium falciparum *genome using a high-density microarray. BMC Genomics 2008 9 398 10.1186/1471-2164-9-398 18724869 PMC2543026

[B23] Tan JC Patel JJ Tan A Blain JC Albert TJ Lobo NF Ferdig MT Optimizing comparative genomic hybridization probes for genotyping and SNP detection in *Plasmodium falciparum*. Genomics 2009 93 543 550 10.1016/j.ygeno.2009.02.007 19285129 PMC3095972

[B24] Aurrecoechea C Brestelli J Brunk BP Dommer J Fischer S Gajria B Gao X Gingle A Grant G Harb OS Heiges M Innamorato F Iodice J Kissinger JC Kraemer E Li W Miller JA Nayak V Pennington C Pinney DF Roos DS Ross C Stoeckert CJ Jr Treatman C Wang H PlasmoDB: a functional genomic database for malaria parasites. Nucleic Acids Res 2009 37 D539 543 10.1093/nar/gkn814 18957442 PMC2686598

[B25] Wong CW Albert TJ Vega VB Norton JE Cutler DJ Richmond TA Stanton LW Liu ET Miller LD Tracking the evolution of the SARS coronavirus using high-throughput, high-density resequencing arrays. Genome Res 2004 14 398 405 10.1101/gr.2141004 14993206 PMC353227

[B26] Neafsey DE Schaffner SF Volkman SK Park D Montgomery P Milner DA Jr Lukens A Rosen D Daniels R Houde N Cortese JF Tyndall E Gates C Stange-Thomann N Sarr O Ndiaye D Ndir O Mboup S Ferreira MU Moraes Sdo L Dash AP Chitnis CE Wiegand RC Hartl DL Birren BW Lander ES Sabeti PC Wirth DF Genome-wide SNP genotyping highlights the role of natural selection in *Plasmodium falciparum *population divergence. Genome Biol 2008 9 R171 10.1186/gb-2008-9-12-r171 19077304 PMC2646275

[B27] Mu J Myers RA Jiang H Liu S Ricklefs S Waisberg M Chotivanich K Wilairatana P Krudsood S White NJ Udomsangpetch R Cui L Ho M Ou F Li H Song J Li G Wang X Seila S Sokunthea S Socheat D Sturdevant DE Porcella SF Fairhurst RM Wellems TE Awadalla P Su XZ *Plasmodium falciparum *genome-wide scans for positive selection, recombination hot spots and resistance to antimalarial drugs. Nat Genet 2010 42 268 271 10.1038/ng.528 20101240 PMC2828519

[B28] Gardner MJ Hall N Fung E White O Berriman M Hyman RW Carlton JM Pain A Nelson KE Bowman S Paulsen IT James K Eisen JA Rutherford K Salzberg SL Craig A Kyes S Chan MS Nene V Shallom SJ Suh B Peterson J Angiuoli S Pertea M Allen J Selengut J Haft D Mather MW Vaidya AB Martin DM Genome sequence of the human malaria parasite *Plasmodium falciparum*. Nature 2002 419 498 511 10.1038/nature01097 12368864 PMC3836256

[B29] Biggs BA Kemp DJ Brown GV Subtelomeric chromosome deletions in field isolates of *Plasmodium falciparum *and their relationship to loss of cytoadherence *in vitro*. Proc Natl Acad Sci USA 1989 86 2428 2432 10.1073/pnas.86.7.2428 2648403 PMC286926

[B30] Day KP Karamalis F Thompson J Barnes DA Peterson C Brown H Brown GV Kemp DJ Genes necessary for expression of a virulence determinant and for transmission of *Plasmodium falciparum *are located on a 0.3-megabase region of chromosome 9. Proc Natl Acad Sci USA 1993 90 8292 8296 10.1073/pnas.90.17.8292 8367496 PMC47335

[B31] Gresham D Curry B Ward A Gordon DB Brizuela L Kruglyak L Botstein D Optimized detection of sequence variation in heterozygous genomes using DNA microarrays with isothermal-melting probes. Proc Natl Acad Sci USA 2010 107 1482 1487 10.1073/pnas.0913883107 20080586 PMC2824413

[B32] Bjornsson HT Albert TJ Ladd-Acosta CM Green RD Rongione MA Middle CM Irizarry RA Broman KW Feinberg AP SNP-specific array-based allele-specific expression analysis. Genome Res 2008 18 771 779 10.1101/gr.073254.107 18369178 PMC2336807

[B33] Chou CC Chen CH Lee TT Peck K Optimization of probe length and the number of probes per gene for optimal microarray analysis of gene expression. Nucleic Acids Res 2004 32 e99 10.1093/nar/gnh099 15243142 PMC484198

[B34] Letowski J Brousseau R Masson L Designing better probes: effect of probe size, mismatch position and number on hybridization in DNA oligonucleotide microarrays. J Microbiol Methods 2004 57 269 278 10.1016/j.mimet.2004.02.002 15063067

[B35] Relogio A Schwager C Richter A Ansorge W Valcarcel J Optimization of oligonucleotide-based DNA microarrays. Nucleic Acids Res 2002 30 e51 10.1093/nar/30.11.e51 12034852 PMC117213

[B36] Selzer RR Richmond TA Pofahl NJ Green RD Eis PS Nair P Brothman AR Stallings RL Analysis of chromosome breakpoints in neuroblastoma at sub-kilobase resolution using fine-tiling oligonucleotide array CGH. Genes Chromosomes Cancer 2005 44 305 319 10.1002/gcc.20243 16075461

[B37] Nuwaysir EF Huang W Albert TJ Singh J Nuwaysir K Pitas A Richmond T Gorski T Berg JP Ballin J McCormick M Norton J Pollock T Sumwalt T Butcher L Porter D Molla M Hall C Blattner F Sussman MR Wallace RL Cerrina F Green RD Gene expression analysis using oligonucleotide arrays produced by maskless photolithography. Genome Res 2002 12 1749 1755 10.1101/gr.362402 12421762 PMC187555

[B38] Singh-Gasson S Green RD Yue Y Nelson C Blattner F Sussman MR Cerrina F Maskless fabrication of light-directed oligonucleotide microarrays using a digital micromirror array. Nat Biotechnol 1999 17 974 978 10.1038/13664 10504697

[B39] MR4/ATCC Methods in Malaria Research 2008 5 Manassas, Virginia

[B40] Trager W Jensen JB Human malaria parasites in continuous culture. Science 1976 193 673 675 10.1126/science.781840 781840

[B41] Samarakoon U Regier A Tan A Desany BA Collins B Tan JC Emrich SJ Ferdig MT High-throughput 454 resequencing for allele discovery and recombination mapping in *Plasmodium falciparum*. BMC Genomics 2011 12 116 10.1186/1471-2164-12-116 21324207 PMC3055840

[B42] Manske HM Kwiatkowski DP SNP-o-matic. Bioinformatics 2009 25 2434 2435 10.1093/bioinformatics/btp403 19574284 PMC2735664

[B43] Nair S Nash D Sudimack D Jaidee A Barends M Uhlemann AC Krishna S Nosten F Anderson TJ Recurrent gene amplification and soft selective sweeps during evolution of multidrug resistance in malaria parasites. Mol Biol Evol 2007 24 562 573 17124182 10.1093/molbev/msl185

