# Supplementary material for: An optimized microarray platform for assaying genomic variation in Plasmodium falciparum field populations
Source: Genome Biol. 2011 Apr 8;12(4):R35. doi: 10.1186/gb-2011-12-4-r35 (PMC3218861; doi:10.1186/gb-2011-12-4-r35)
Supplement: Additional file 1 — Supplementary Figures S1 to S3. Figure S1: pptimal probe melting temperature is consistent in exons, introns, and intergenic regions. Mean Dscore is plotted by probe melting temperature in exons, introns, and intergenic regions; vertical lines indicate 95% confidence intervals. Probes with approximately 66°C melting temperature consistently provided the best performance. Figure S2: CGH data reproducibility. CGH scatterplots for individual CNV events are displayed for replicate hybridizations from independent labeling reactions demonstrating data reproducibility. CNV events from three separate parasite clones are displayed: (a) Dd2; (b) HB3; (c) SC05. The CNV breakpoints are precisely identified between hybridizations. Figure S3: CNV detection in a WGA field sample. CGH scatterplot for a CNV event detected in a WGA field sample, M1064. Four genes (PFE1150w, PFE1155c, PFE1160w, and PFE1165c) are affected by this CNV, including the P. falciparum multidrug resistance gene, pfmdr1. [file gb-2011-12-4-r35-S1.PDF]

Supplementary Figure S1

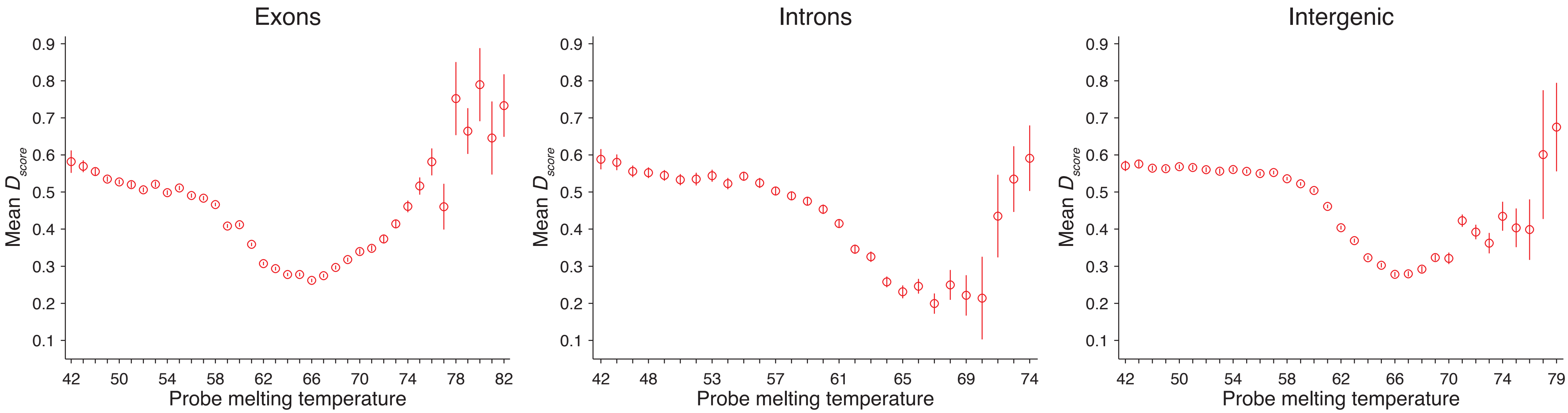

Optimal probe melting temperature is consistent in exons, introns, and intergenic regions

Mean D_{score} is plotted by probe melting temperature in exons, introns, and intergenic regions; vertical lines indicate 95% confidence intervals. Probes with an approximately 66°C melting temperature consistently provided the best performance.

Supplementary Figure S2

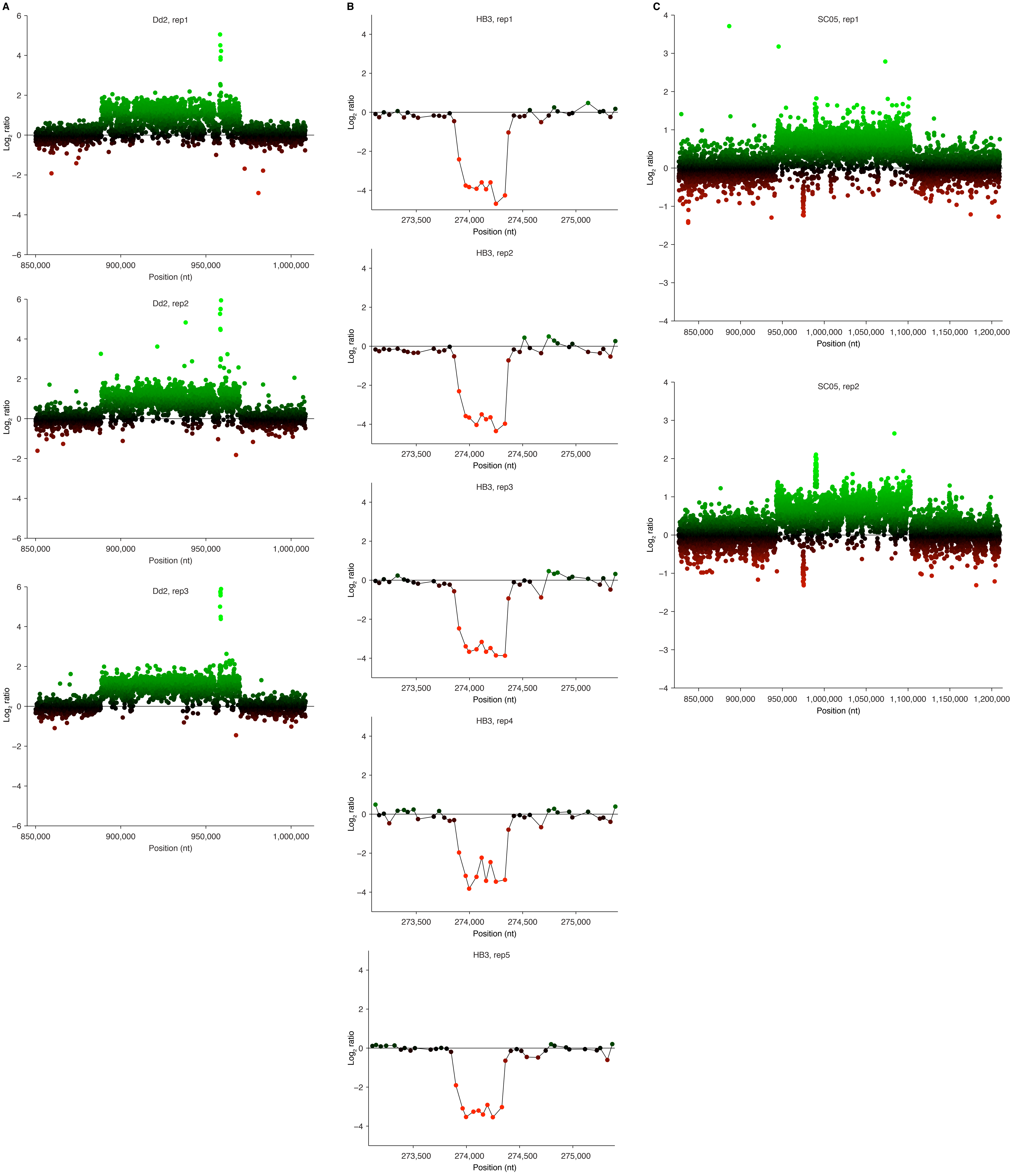

CGH data reproducibility

CGH scatterplots for individual CNV events are displayed for replicate hybridizations from independent labeling reactions demonstrating data reproducibility.
CNV events from three separate parasite clones are displayed: (A) Dd2; (B) HB3; (C) SC05.
The CNV breakpoints are precisely identified between hybridizations.

Supplementary Figure S3

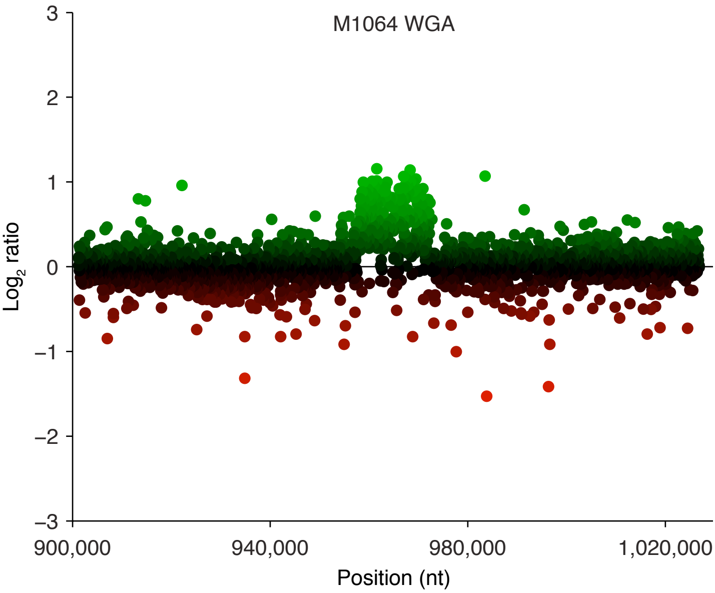

CNV detection in a WGA field sample
CGH scatterplot for a CNV event detected in a WGA field sample, M1064. Four genes (PFE1150w, PFE1155c, PFE1160w, and PFE1165c) are affected by this CNV including the *P. falciparum* multidrug resistance gene, *pfmdr1*.
